# (*E*)-3-(2-Eth­oxy­phen­yl)-1-{4-[(2-fluoro­phen­yl)(4-fluoro­phen­yl)meth­yl]piperazin-1-yl}prop-2-en-1-one

**DOI:** 10.1107/S1600536812024130

**Published:** 2012-05-31

**Authors:** Yan Zhong, Bin Wu

**Affiliations:** a School of Chemistry and Chemical Engineering, Southeast University, Sipailou No. 2 Nanjing, Nanjing 210096, People’s Republic of China; b School of Pharmacy, Nanjing Medical University, Hanzhong Road No. 140 Nanjing, Nanjing 210029, People’s Republic of China

## Abstract

In the title compound, C_28_H_28_F_2_N_2_O_2_, the piperazine ring has a chair conformation with the pendant N—C bonds in equatorial orientations. The C=C double bond has an *E* conformation and the dihedral angle between the fluoro­benzene rings is 70.8 (3)°. In the crystal, mol­ecules are linked by C—H⋯O and C—H⋯F hydrogen bonds.

## Related literature

For a related structure and background to cinnamic acid derivatives, see: Teng *et al.* (2011 ▶); Zhong *et al.* (2012 ▶). For further synthetic details, see: Wu *et al.* (2008 ▶).
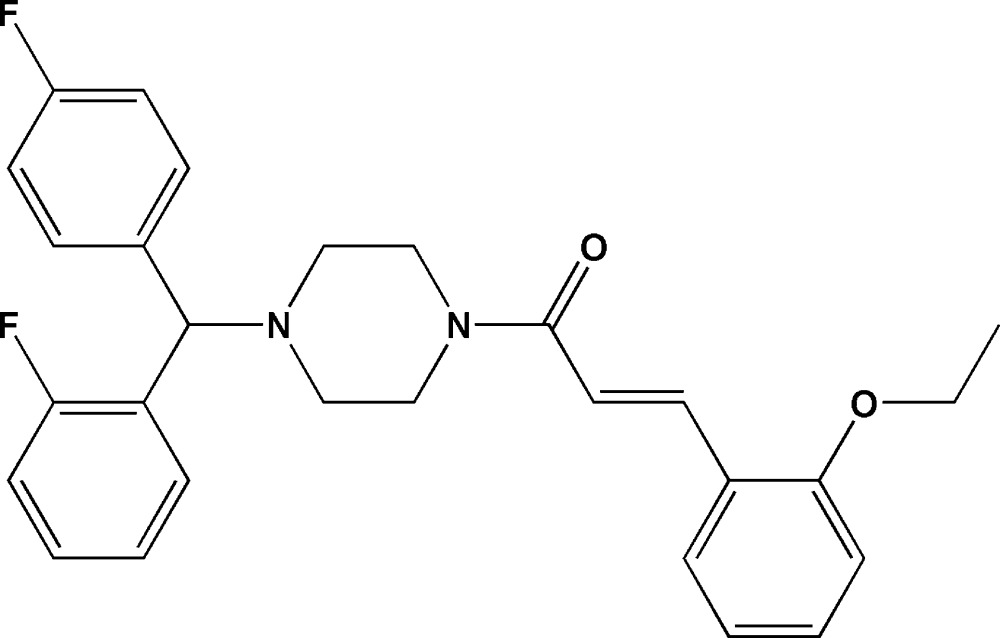


## Experimental

### 

#### Crystal data

C_28_H_28_F_2_N_2_O_2_
*M* *_r_* = 462.52
Orthorhombic, 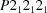

*a* = 8.8550 (18) Å
*b* = 12.827 (3) Å
*c* = 22.432 (5) Å
*V* = 2547.9 (9) Å^3^
*Z* = 4
Mo *K*α radiation
μ = 0.09 mm^−1^
*T* = 293 K
0.30 × 0.20 × 0.10 mm

#### Data collection

Enraf–Nonius CAD-4 diffractometer
Absorption correction: ψ scan (North *et al.*, 1968 ▶) *T*_min_ = 0.975, *T*_max_ = 0.992
5217 measured reflections
2677 independent reflections
1328 reflections with *I* > 2σ(*I*)
*R*_int_ = 0.092
3 standard reflections every 200 reflections intensity decay: 1%

#### Refinement

*R*[*F* ^2^ > 2σ(*F* ^2^)] = 0.063
*wR*(*F* ^2^) = 0.160
*S* = 1.00
2677 reflections
307 parameters
1 restraint
H-atom parameters constrained
Δρ_max_ = 0.23 e Å^−3^
Δρ_min_ = −0.12 e Å^−3^

### 

Data collection: *CAD-4 EXPRESS* (Enraf–Nonius, 1989 ▶); cell refinement: *CAD-4 EXPRESS*; data reduction: *XCAD4* (Harms & Wocadlo, 1995 ▶); program(s) used to solve structure: *SHELXS97* (Sheldrick, 2008 ▶); program(s) used to refine structure: *SHELXL97* (Sheldrick, 2008 ▶); molecular graphics: *SHELXTL* (Sheldrick, 2008 ▶); software used to prepare material for publication: *SHELXL97*.

## Supplementary Material

Crystal structure: contains datablock(s) I, global. DOI: 10.1107/S1600536812024130/hb6812sup1.cif

Structure factors: contains datablock(s) I. DOI: 10.1107/S1600536812024130/hb6812Isup2.hkl

Supplementary material file. DOI: 10.1107/S1600536812024130/hb6812Isup3.cml

Additional supplementary materials:  crystallographic information; 3D view; checkCIF report

## Figures and Tables

**Table 1:** Hydrogen-bond geometry (Å, °)

*D*—H⋯*A*	*D*—H	H⋯*A*	*D*⋯*A*	*D*—H⋯*A*
C5—H5*A*⋯O1^i^	0.93	2.44	3.316 (6)	156
C15—H15*A*⋯F2^ii^	0.97	2.36	3.241 (6)	150
C25—H25*A*⋯F1^iii^	0.93	2.55	3.166 (7)	124

Symmetry codes: (i) 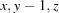; (ii) 
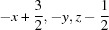
; (iii) 
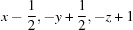
.

**Fig. 1.**
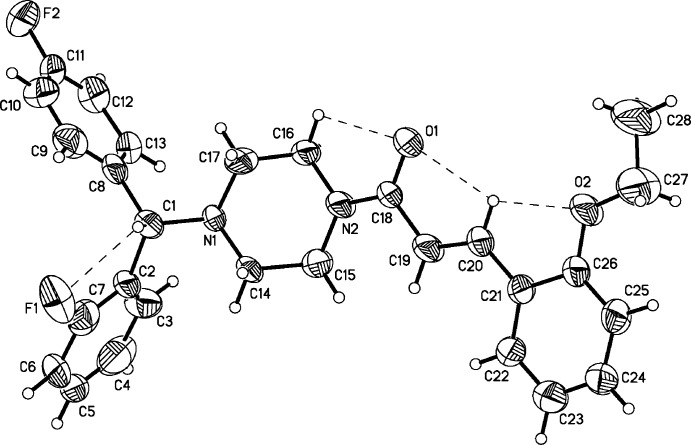
The molecular structure of (I) with displacement ellipsoids for non-H drawn at 70% probability level.

**Fig. 2.**
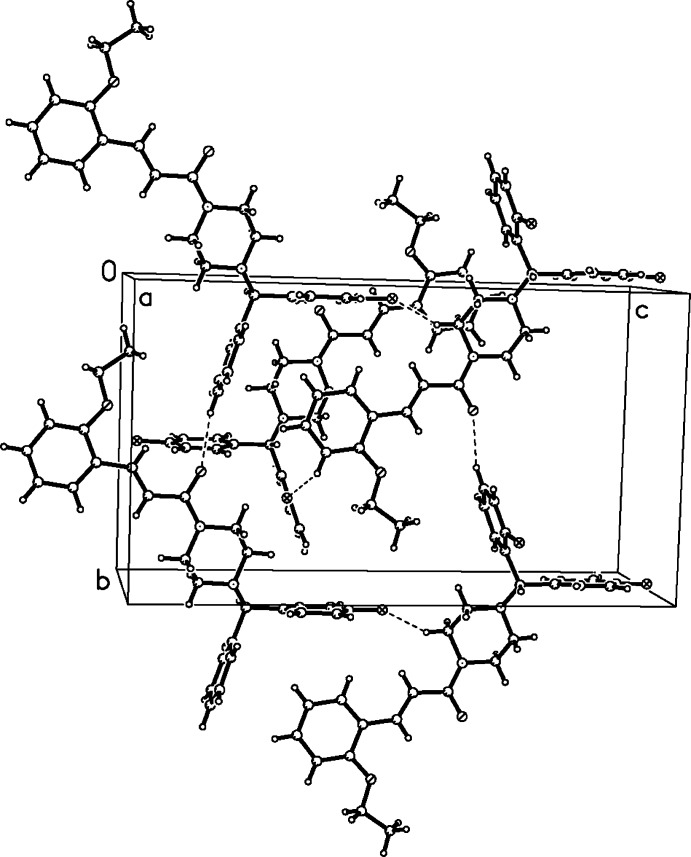
Packing diagram of the title compound.

## supplementary crystallographic information

### Comment

As a continuation of our study of cinnamic acid derivatives (Teng *et al.*, 2011; Zhong *et al.*, 2012), we present here the title compound (I). In (I) (Fig. 1), all bond lengths and angles are normal and correspond to those observed in related compounds (Teng *et al.*, 2011; Zhong *et al.*, 2012). The molecule of (I) exists an E configulation with respect to the C19=C20 ethene bond [1.296 (6)]. The piperazine ring adopts a chair conformation with puchering parameters Q = 0.498 (6), Theta = 8.6 (6), Phi = 136 (4). The molecular structure is stabilized by intramolecular C—H···O and C—H···F hydrogen bonds. In the crystal, molecules are linked by intermolecular C—H···O and C—H···F hydrogen bonds.

### Experimental

The synthesis follows the method of Wu *et al.* (2008). The title compound was prepared by stirring a mixture of (*E*)-3-(2-ethoxyphenyl)acrylic acid (0.769 g; 4 mmol), thionyl chloride (2 ml) and dichloromethane (30 ml) for 6 h at room temperature. The solvent was removed under reduced pressure. The residue was dissolved in acetone (15 ml) and reacted with 1-((2-fluorophenyl)(4-fluorophenyl)methyl)piperazine (1.730 g; 6 mmol) in the presence of triethylamine (5 ml) for 12 h at room temperature. The resultant mixture was cooled. The solid, (*E*)-3-(2-ethoxyphenyl)-1-(4-((2- fluorophenyl)(4-fluorophenyl)methyl)piperazin-1-yl)prop-2-en-1-one obtained was filtered and was recrystallized from ethanol. The colorless single crystals of the title compound used in *x*-ray diffraction studies were grown in ethanol by a slow evaporation at room temperature.

### Refinement

The absolution structure was indeterminate in the present experiment and Friedel pairs were merged. The arbitrarily assigined chirality of the stereogenic centre is C1 S*. All non-hydrogen atoms were refined anisotropically. All hydrogen atoms were positioned geometrically with C—H distances ranging from 0.93 Å to 0.98 Å and refined as riding on their parent atoms with *U*ĩso~(H) = 1.2 or 1.5*U*~eq~ of the carrier atom.

### Crystal data

**Table:**

C_28_H_28_F_2_N_2_O_2_	*D*_x_ = 1.206 Mg m^−^^3^
*M**_r_* = 462.52	Mo *K*α radiation, λ = 0.71073 Å
Orthorhombic, *P*2_1_2_1_2_1_	Cell parameters from 25 reflections
*a* = 8.8550 (18) Å	θ = 9–13°
*b* = 12.827 (3) Å	µ = 0.09 mm^−^^1^
*c* = 22.432 (5) Å	*T* = 293 K
*V* = 2547.9 (9) Å^3^	Block, colorless
*Z* = 4	0.30 × 0.20 × 0.10 mm
*F*(000) = 976	

### Data collection

**Table:**

Enraf–Nonius CAD-4 diffractometer	1328 reflections with *I* > 2σ(*I*)
Radiation source: fine-focus sealed tube	*R*_int_ = 0.092
Graphite monochromator	θ_max_ = 25.5°, θ_min_ = 1.8°
ω/2θ scans	*h* = 0→10
Absorption correction: ψ scan (North *et al.*, 1968)	*k* = 0→15
*T*_min_ = 0.975, *T*_max_ = 0.992	*l* = −27→27
5217 measured reflections	3 standard reflections every 200 reflections
2677 independent reflections	intensity decay: 1%

### Refinement

**Table:**

Refinement on *F*^2^	Primary atom site location: structure-invariant direct methods
Least-squares matrix: full	Secondary atom site location: difference Fourier map
*R*[*F*^2^ > 2σ(*F*^2^)] = 0.063	Hydrogen site location: inferred from neighbouring sites
*wR*(*F*^2^) = 0.160	H-atom parameters constrained
*S* = 1.00	*w* = 1/[σ^2^(*F*_o_^2^) + (0.063*P*)^2^] where *P* = (*F*_o_^2^ + 2*F*_c_^2^)/3
2677 reflections	(Δ/σ)_max_ < 0.001
307 parameters	Δρ_max_ = 0.23 e Å^−^^3^
1 restraint	Δρ_min_ = −0.12 e Å^−^^3^

### Special details

**Table:**

Geometry. All e.s.d.'s (except the e.s.d. in the dihedral angle between two l.s. planes) are estimated using the full covariance matrix. The cell e.s.d.'s are taken into account individually in the estimation of e.s.d.'s in distances, angles and torsion angles; correlations between e.s.d.'s in cell parameters are only used when they are defined by crystal symmetry. An approximate (isotropic) treatment of cell e.s.d.'s is used for estimating e.s.d.'s involving l.s. planes.
Refinement. Refinement of *F*^2^ against ALL reflections. The weighted *R*-factor *wR* and goodness of fit *S* are based on *F*^2^, conventional *R*-factors *R* are based on *F*, with *F* set to zero for negative *F*^2^. The threshold expression of *F*^2^ > σ(*F*^2^) is used only for calculating *R*-factors(gt) *etc*. and is not relevant to the choice of reflections for refinement. *R*-factors based on *F*^2^ are statistically about twice as large as those based on *F*, and *R*- factors based on ALL data will be even larger.

### Fractional atomic coordinates and isotropic or equivalent isotropic displacement parameters (Å^2^)

**Table:**

	*x*	*y*	*z*	*U*_iso_*/*U*_eq_	
O1	0.7258 (5)	0.3977 (2)	0.63108 (15)	0.0891 (11)	
N1	0.7411 (6)	0.0370 (2)	0.69576 (16)	0.0739 (10)	
F1	1.0297 (5)	−0.2050 (3)	0.6989 (2)	0.1490 (16)	
C1	0.8192 (7)	−0.0465 (4)	0.7234 (2)	0.0846 (16)	
H1A	0.9274	−0.0383	0.7154	0.102*	
O2	0.7460 (6)	0.6004 (3)	0.46140 (16)	0.1160 (13)	
F2	0.7518 (6)	−0.0447 (3)	0.97218 (14)	0.1447 (15)	
N2	0.6907 (7)	0.2261 (3)	0.63068 (19)	0.113 (2)	
C2	0.7675 (8)	−0.1522 (3)	0.6976 (2)	0.0780 (15)	
C3	0.6258 (10)	−0.1824 (6)	0.6896 (3)	0.109 (2)	
H3A	0.5466	−0.1367	0.6972	0.131*	
C4	0.5969 (12)	−0.2819 (9)	0.6698 (3)	0.140 (3)	
H4A	0.4976	−0.3038	0.6647	0.168*	
C5	0.7119 (15)	−0.3482 (4)	0.6578 (3)	0.132 (4)	
H5A	0.6893	−0.4150	0.6443	0.159*	
C6	0.8589 (12)	−0.3209 (8)	0.6649 (4)	0.146 (4)	
H6A	0.9384	−0.3656	0.6559	0.175*	
C7	0.8803 (9)	−0.2219 (7)	0.6862 (3)	0.105 (2)	
C8	0.7955 (9)	−0.0479 (4)	0.7912 (3)	0.0894 (18)	
C9	0.9141 (8)	−0.0567 (5)	0.8273 (4)	0.105 (2)	
H9A	1.0092	−0.0654	0.8104	0.126*	
C10	0.9031 (10)	−0.0537 (6)	0.8849 (4)	0.111 (2)	
H10A	0.9901	−0.0560	0.9081	0.133*	
C11	0.7695 (11)	−0.0476 (4)	0.9110 (3)	0.0878 (16)	
C12	0.6397 (8)	−0.0441 (5)	0.8813 (3)	0.1040 (19)	
H12A	0.5471	−0.0432	0.9008	0.125*	
C13	0.6508 (8)	−0.0417 (5)	0.8162 (3)	0.0949 (18)	
H13A	0.5652	−0.0363	0.7924	0.114*	
C14	0.7528 (7)	0.0413 (3)	0.6316 (2)	0.0761 (13)	
H14A	0.7235	−0.0256	0.6152	0.091*	
H14B	0.8574	0.0536	0.6208	0.091*	
C15	0.6571 (8)	0.1243 (4)	0.6045 (2)	0.101 (2)	
H15A	0.6751	0.1268	0.5618	0.121*	
H15B	0.5514	0.1077	0.6108	0.121*	
C16	0.6743 (8)	0.2221 (4)	0.6953 (2)	0.106 (2)	
H16A	0.7091	0.2873	0.7124	0.127*	
H16B	0.5684	0.2140	0.7053	0.127*	
C17	0.7575 (8)	0.1385 (3)	0.7205 (2)	0.0914 (17)	
H17A	0.7310	0.1344	0.7624	0.110*	
H17B	0.8637	0.1568	0.7184	0.110*	
C18	0.7198 (8)	0.3163 (4)	0.6033 (2)	0.0939 (19)	
C19	0.7188 (7)	0.3198 (4)	0.5371 (2)	0.0975 (19)	
H19A	0.7115	0.2575	0.5160	0.117*	
C20	0.7279 (7)	0.4064 (3)	0.5076 (2)	0.0801 (14)	
H20A	0.7312	0.4669	0.5304	0.096*	
C21	0.7336 (6)	0.4210 (3)	0.4434 (2)	0.0707 (12)	
C22	0.7270 (9)	0.3415 (4)	0.4028 (2)	0.102 (2)	
H22A	0.7167	0.2738	0.4170	0.123*	
C23	0.7348 (8)	0.3568 (5)	0.3411 (3)	0.1042 (17)	
H23A	0.7331	0.3005	0.3150	0.125*	
C24	0.7448 (7)	0.4536 (5)	0.3208 (2)	0.0986 (17)	
H24A	0.7495	0.4650	0.2799	0.118*	
C25	0.7485 (7)	0.5382 (4)	0.3591 (2)	0.0975 (16)	
H25A	0.7540	0.6055	0.3438	0.117*	
C26	0.7439 (7)	0.5228 (4)	0.4206 (2)	0.0794 (13)	
C27	0.7553 (18)	0.7032 (5)	0.4448 (3)	0.213 (5)	
H27A	0.6688	0.7217	0.4205	0.255*	
H27B	0.8461	0.7145	0.4214	0.255*	
C28	0.759 (2)	0.7692 (5)	0.4994 (5)	0.269 (8)	
H28A	0.7702	0.8410	0.4882	0.403*	
H28B	0.8428	0.7487	0.5239	0.403*	
H28C	0.6667	0.7603	0.5212	0.403*	

### Atomic displacement parameters (Å^2^)

**Table:**

	*U*^11^	*U*^22^	*U*^33^	*U*^12^	*U*^13^	*U*^23^
O1	0.134 (3)	0.0448 (16)	0.088 (2)	−0.009 (2)	−0.015 (3)	0.0009 (17)
N1	0.129 (3)	0.0314 (16)	0.061 (2)	0.001 (3)	−0.015 (3)	0.0062 (15)
F1	0.148 (3)	0.102 (3)	0.197 (4)	0.038 (3)	0.015 (3)	0.023 (3)
C1	0.126 (4)	0.059 (3)	0.069 (3)	0.017 (3)	0.009 (3)	−0.001 (3)
O2	0.209 (4)	0.0492 (18)	0.090 (2)	0.002 (3)	0.014 (4)	0.0069 (18)
F2	0.249 (4)	0.117 (2)	0.068 (2)	0.020 (4)	−0.015 (3)	0.0211 (18)
N2	0.238 (6)	0.0337 (19)	0.067 (3)	0.002 (3)	−0.012 (3)	0.0035 (19)
C2	0.127 (5)	0.042 (2)	0.065 (3)	0.003 (4)	0.000 (4)	0.004 (2)
C3	0.145 (6)	0.099 (5)	0.083 (4)	0.012 (5)	0.009 (5)	−0.019 (4)
C4	0.189 (8)	0.134 (8)	0.098 (6)	−0.044 (7)	−0.042 (5)	0.021 (6)
C5	0.288 (13)	0.036 (3)	0.072 (4)	0.000 (6)	−0.044 (7)	0.007 (3)
C6	0.217 (10)	0.113 (7)	0.108 (6)	0.111 (7)	−0.026 (7)	−0.020 (5)
C7	0.127 (5)	0.120 (6)	0.068 (4)	0.027 (5)	0.016 (4)	0.018 (4)
C8	0.152 (6)	0.041 (2)	0.075 (4)	0.015 (3)	0.004 (4)	0.011 (2)
C9	0.119 (5)	0.071 (4)	0.124 (7)	−0.006 (4)	−0.011 (5)	−0.008 (4)
C10	0.152 (7)	0.094 (5)	0.088 (5)	−0.003 (5)	−0.026 (5)	−0.015 (4)
C11	0.131 (5)	0.057 (3)	0.076 (4)	0.007 (4)	−0.019 (5)	0.013 (3)
C12	0.121 (5)	0.080 (4)	0.110 (6)	0.007 (4)	−0.006 (5)	0.008 (4)
C13	0.129 (5)	0.068 (3)	0.087 (5)	0.028 (4)	−0.029 (4)	0.002 (4)
C14	0.124 (4)	0.040 (2)	0.064 (3)	0.016 (3)	0.010 (3)	−0.001 (2)
C15	0.176 (6)	0.061 (3)	0.066 (3)	−0.004 (4)	−0.013 (3)	0.002 (3)
C16	0.211 (7)	0.055 (3)	0.050 (3)	0.017 (4)	−0.006 (4)	−0.001 (3)
C17	0.163 (5)	0.045 (2)	0.066 (3)	−0.042 (4)	−0.015 (4)	−0.001 (2)
C18	0.172 (6)	0.041 (2)	0.069 (3)	0.016 (4)	−0.025 (4)	−0.002 (2)
C19	0.173 (6)	0.047 (2)	0.073 (3)	0.001 (4)	0.010 (4)	0.001 (2)
C20	0.120 (4)	0.047 (2)	0.073 (3)	0.000 (3)	−0.001 (3)	0.010 (2)
C21	0.089 (3)	0.056 (3)	0.067 (3)	−0.007 (3)	−0.003 (3)	0.007 (2)
C22	0.162 (6)	0.062 (3)	0.083 (4)	−0.001 (4)	−0.038 (5)	−0.002 (3)
C23	0.135 (5)	0.084 (4)	0.094 (4)	0.002 (4)	0.001 (5)	−0.008 (3)
C24	0.121 (4)	0.095 (4)	0.080 (3)	0.027 (5)	0.011 (4)	−0.006 (3)
C25	0.136 (4)	0.078 (3)	0.078 (3)	0.006 (5)	0.015 (4)	0.025 (3)
C26	0.106 (4)	0.060 (3)	0.072 (3)	0.007 (4)	0.001 (4)	0.009 (2)
C27	0.456 (17)	0.060 (4)	0.122 (6)	−0.052 (9)	−0.010 (10)	0.022 (4)
C28	0.60 (2)	0.067 (4)	0.137 (7)	−0.016 (10)	−0.001 (13)	−0.008 (5)

### Geometric parameters (Å, º)

**Table:**

O1—C18	1.216 (5)	C13—H13A	0.9300
N1—C1	1.417 (6)	C14—C15	1.490 (7)
N1—C17	1.423 (5)	C14—H14A	0.9700
N1—C14	1.444 (6)	C14—H14B	0.9700
F1—C7	1.371 (8)	C15—H15A	0.9700
C1—C8	1.536 (7)	C15—H15B	0.9700
C1—C2	1.545 (7)	C16—C17	1.417 (7)
C1—H1A	0.9800	C16—H16A	0.9700
O2—C26	1.351 (6)	C16—H16B	0.9700
O2—C27	1.374 (7)	C17—H17A	0.9700
F2—C11	1.383 (7)	C17—H17B	0.9700
N2—C18	1.335 (7)	C18—C19	1.488 (7)
N2—C16	1.458 (6)	C19—C20	1.296 (6)
N2—C15	1.463 (7)	C19—H19A	0.9300
C2—C3	1.325 (9)	C20—C21	1.452 (6)
C2—C7	1.365 (9)	C20—H20A	0.9300
C3—C4	1.375 (12)	C21—C22	1.368 (7)
C3—H3A	0.9300	C21—C26	1.406 (6)
C4—C5	1.354 (13)	C22—C23	1.401 (7)
C4—H4A	0.9300	C22—H22A	0.9300
C5—C6	1.358 (12)	C23—C24	1.325 (8)
C5—H5A	0.9300	C23—H23A	0.9300
C6—C7	1.370 (12)	C24—C25	1.385 (7)
C6—H6A	0.9300	C24—H24A	0.9300
C8—C9	1.331 (8)	C25—C26	1.394 (7)
C8—C13	1.401 (9)	C25—H25A	0.9300
C9—C10	1.296 (8)	C27—C28	1.489 (11)
C9—H9A	0.9300	C27—H27A	0.9700
C10—C11	1.323 (9)	C27—H27B	0.9700
C10—H10A	0.9300	C28—H28A	0.9600
C11—C12	1.328 (8)	C28—H28B	0.9600
C12—C13	1.463 (8)	C28—H28C	0.9600
C12—H12A	0.9300		
			
C1—N1—C17	118.1 (4)	N2—C15—H15A	109.5
C1—N1—C14	115.4 (4)	C14—C15—H15A	109.5
C17—N1—C14	110.2 (3)	N2—C15—H15B	109.5
N1—C1—C8	112.0 (4)	C14—C15—H15B	109.5
N1—C1—C2	110.8 (4)	H15A—C15—H15B	108.0
C8—C1—C2	108.7 (4)	C17—C16—N2	111.8 (5)
N1—C1—H1A	108.4	C17—C16—H16A	109.3
C8—C1—H1A	108.4	N2—C16—H16A	109.3
C2—C1—H1A	108.4	C17—C16—H16B	109.3
C26—O2—C27	121.6 (5)	N2—C16—H16B	109.3
C18—N2—C16	120.4 (4)	H16A—C16—H16B	107.9
C18—N2—C15	129.0 (4)	C16—C17—N1	118.9 (5)
C16—N2—C15	110.4 (4)	C16—C17—H17A	107.6
C3—C2—C7	118.5 (6)	N1—C17—H17A	107.6
C3—C2—C1	126.0 (6)	C16—C17—H17B	107.6
C7—C2—C1	115.3 (6)	N1—C17—H17B	107.6
C2—C3—C4	119.4 (7)	H17A—C17—H17B	107.0
C2—C3—H3A	120.3	O1—C18—N2	121.2 (4)
C4—C3—H3A	120.3	O1—C18—C19	119.1 (4)
C5—C4—C3	120.5 (9)	N2—C18—C19	118.9 (4)
C5—C4—H4A	119.7	C20—C19—C18	122.4 (5)
C3—C4—H4A	119.7	C20—C19—H19A	118.8
C4—C5—C6	122.4 (8)	C18—C19—H19A	118.8
C4—C5—H5A	118.8	C19—C20—C21	128.2 (5)
C6—C5—H5A	118.8	C19—C20—H20A	115.9
C5—C6—C7	114.4 (7)	C21—C20—H20A	115.9
C5—C6—H6A	122.8	C22—C21—C26	116.9 (4)
C7—C6—H6A	122.8	C22—C21—C20	124.2 (4)
C2—C7—C6	124.8 (8)	C26—C21—C20	118.9 (4)
C2—C7—F1	124.3 (7)	C21—C22—C23	123.5 (5)
C6—C7—F1	110.7 (8)	C21—C22—H22A	118.3
C9—C8—C13	118.9 (6)	C23—C22—H22A	118.3
C9—C8—C1	119.7 (7)	C24—C23—C22	118.3 (6)
C13—C8—C1	121.4 (6)	C24—C23—H23A	120.8
C10—C9—C8	123.0 (7)	C22—C23—H23A	120.8
C10—C9—H9A	118.5	C23—C24—C25	121.5 (5)
C8—C9—H9A	118.5	C23—C24—H24A	119.3
C9—C10—C11	120.7 (7)	C25—C24—H24A	119.3
C9—C10—H10A	119.7	C24—C25—C26	120.2 (5)
C11—C10—H10A	119.7	C24—C25—H25A	119.9
C10—C11—C12	123.6 (6)	C26—C25—H25A	119.9
C10—C11—F2	122.9 (7)	O2—C26—C25	124.4 (4)
C12—C11—F2	113.5 (8)	O2—C26—C21	116.0 (4)
C11—C12—C13	116.3 (6)	C25—C26—C21	119.6 (5)
C11—C12—H12A	121.9	O2—C27—C28	108.9 (6)
C13—C12—H12A	121.9	O2—C27—H27A	109.9
C8—C13—C12	117.4 (6)	C28—C27—H27A	109.9
C8—C13—H13A	121.3	O2—C27—H27B	109.9
C12—C13—H13A	121.3	C28—C27—H27B	109.9
N1—C14—C15	113.2 (4)	H27A—C27—H27B	108.3
N1—C14—H14A	108.9	C27—C28—H28A	109.5
C15—C14—H14A	108.9	C27—C28—H28B	109.5
N1—C14—H14B	108.9	H28A—C28—H28B	109.5
C15—C14—H14B	108.9	C27—C28—H28C	109.5
H14A—C14—H14B	107.8	H28A—C28—H28C	109.5
N2—C15—C14	110.9 (5)	H28B—C28—H28C	109.5
			
C17—N1—C1—C8	48.9 (7)	C1—N1—C14—C15	174.5 (5)
C14—N1—C1—C8	−177.6 (5)	C17—N1—C14—C15	−48.4 (7)
C17—N1—C1—C2	170.4 (5)	C18—N2—C15—C14	130.6 (7)
C14—N1—C1—C2	−56.1 (6)	C16—N2—C15—C14	−55.2 (8)
N1—C1—C2—C3	−47.6 (8)	N1—C14—C15—N2	55.0 (7)
C8—C1—C2—C3	75.9 (7)	C18—N2—C16—C17	−133.4 (7)
N1—C1—C2—C7	137.0 (5)	C15—N2—C16—C17	51.8 (8)
C8—C1—C2—C7	−99.5 (6)	N2—C16—C17—N1	−49.8 (9)
C7—C2—C3—C4	−0.4 (10)	C1—N1—C17—C16	−176.9 (6)
C1—C2—C3—C4	−175.6 (6)	C14—N1—C17—C16	47.4 (8)
C2—C3—C4—C5	−0.9 (11)	C16—N2—C18—O1	−3.7 (11)
C3—C4—C5—C6	0.4 (12)	C15—N2—C18—O1	170.0 (7)
C4—C5—C6—C7	1.3 (12)	C16—N2—C18—C19	−173.2 (6)
C3—C2—C7—C6	2.4 (10)	C15—N2—C18—C19	0.5 (11)
C1—C2—C7—C6	178.1 (7)	O1—C18—C19—C20	1.8 (11)
C3—C2—C7—F1	−172.3 (6)	N2—C18—C19—C20	171.5 (7)
C1—C2—C7—F1	3.4 (8)	C18—C19—C20—C21	177.6 (6)
C5—C6—C7—C2	−2.8 (12)	C19—C20—C21—C22	1.4 (11)
C5—C6—C7—F1	172.5 (7)	C19—C20—C21—C26	−179.8 (7)
N1—C1—C8—C9	−131.1 (6)	C26—C21—C22—C23	2.1 (11)
C2—C1—C8—C9	106.2 (7)	C20—C21—C22—C23	−179.0 (7)
N1—C1—C8—C13	49.9 (7)	C21—C22—C23—C24	−2.0 (13)
C2—C1—C8—C13	−72.9 (7)	C22—C23—C24—C25	0.4 (12)
C13—C8—C9—C10	−4.2 (10)	C23—C24—C25—C26	0.9 (11)
C1—C8—C9—C10	176.8 (6)	C27—O2—C26—C25	−1.0 (12)
C8—C9—C10—C11	3.9 (12)	C27—O2—C26—C21	−179.8 (9)
C9—C10—C11—C12	0.0 (11)	C24—C25—C26—O2	−179.6 (6)
C9—C10—C11—F2	179.7 (6)	C24—C25—C26—C21	−0.8 (11)
C10—C11—C12—C13	−3.2 (9)	C22—C21—C26—O2	178.2 (6)
F2—C11—C12—C13	177.1 (5)	C20—C21—C26—O2	−0.7 (9)
C9—C8—C13—C12	0.7 (9)	C22—C21—C26—C25	−0.7 (10)
C1—C8—C13—C12	179.8 (5)	C20—C21—C26—C25	−179.6 (6)
C11—C12—C13—C8	2.7 (9)	C26—O2—C27—C28	−178.7 (11)

### Hydrogen-bond geometry (Å, º)

**Table:**

*D*—H···*A*	*D*—H	H···*A*	*D*···*A*	*D*—H···*A*
C5—H5*A*···O1^i^	0.93	2.44	3.316 (6)	156
C15—H15*A*···F2^ii^	0.97	2.36	3.241 (6)	150
C25—H25*A*···F1^iii^	0.93	2.55	3.166 (7)	124

Symmetry codes: (i) *x*, *y*−1, *z*; (ii) −*x*+3/2, −*y*, *z*−1/2; (iii) *x*−1/2, −*y*+1/2, −*z*+1.
